# Supplementary material for: Feasibility and readiness to implement Robson classification to monitor caesarean sections in public hospitals in Myanmar: Formative research
Source: PLOS Glob Public Health. 2023 Jan 30;3(1):e0001388. doi: 10.1371/journal.pgph.0001388 (PMC10022350; doi:10.1371/journal.pgph.0001388)
Supplement: S1 Text — (DOCX) [file pgph.0001388.s002.docx]

*Thank you for agreeing to participate in this study. Now I would like to speak to you about interventions that could reduce unnecessary Caesarean sections. We would like your help to identify solutions for optimizing the use of Caesarean section in your setting.*

1. *In order to understand drivers of rising Caesarean section rates, we need to have tools to monitor and compare CS rates in a setting over time. One way to do this is the Robson classification system, which prospectively classifies women admitted for delivery into one of ten groups. To implement the Robson classification system, we need the following data points: parity, previous caesarean section, onset of labour, gestational age, fetal presentation/lie and number of foetuses from medical records.*
   1. Are these data routinely collected in your setting?
   2. Do you foresee any challenges with incomplete medical records complicating data collection?
   3. What can be done to ensure that this data is routinely captured for every woman when she is admitted for childbirth?
   4. What type of person would be the most appropriate facility-level champion to implement Robson classification system?
   5. Who is the best person to *record* data for Robson classification?
   6. Who is the best person to *analyze* and report on Robson classification data_
   7. As a [provider/administrator], how often would you like to see this data (for example, weekly or monthly?)
