# Supplementary material for: Feasibility and readiness to implement Robson classification to monitor caesarean sections in public hospitals in Myanmar: Formative research
Source: PLOS Glob Public Health. 2023 Jan 30;3(1):e0001388. doi: 10.1371/journal.pgph.0001388 (PMC10022350; doi:10.1371/journal.pgph.0001388)
Supplement: S2 Text — (DOCX) [file pgph.0001388.s003.docx]

**Appendix 2. Labour ward observation and medical record assessment**

*For each of the study health facilities, please have a member of the research team visit to conduct an observation of the labour ward and medical records. This activity is part of the readiness assessment, to explore factors to be assessed, considered and integrated into implementation plans.* *Assessment of facility and medical records systems for implementation of Robson classification will be conducted.*

**Assessment of facility and medical records systems for implementation of Robson classification**

*Please review individual medical records to assess the information currently collected related to key obstetric variables at an individual level. Please also review any facility-level register, log book or other records to assess information currently collected related to key obstetric variables at a facility level. It may be helpful to discuss the medical and facility records with the staff, e.g.: a matron-in-charge or head of obstetrics. Collecting this information will help to inform the implementation of the Robson classification system, e.g.: to identify what data is already routinely collected, and what data may need to be added to routine data collection.*

Description of key obstetric variables that are currently included on an individual’s medical record (parity, previous caesarean section, onset of labour, gestational age, fetal presentation or lie, number of foetuses).

|  |
| --- |

Description of the consistency of reporting for these indicators (e.g.: consistently reported across all records reviewed, some data missing – be specific).

|  |
| --- |

Review of medical records to assess if key obstetric variables needed for Robson classification are correctly and consistently reported at an individual level:

*Parity*

|  |
| --- |

*Previous caesarean section*

|  |
| --- |

*Onset of labour (spontaneous, induced, no labour/pre-labour caesarean section)*

|  |
| --- |

*Gestational age (preterm <37 weeks, term > 37 weeks)*

|  |
| --- |

*Fetal presentation or lie (cephalic, breech, transverse)*

|  |
| --- |

*Number of fetuses (singleton, multiple)*

|  |
| --- |

Who is responsible for completing the individual-level medical records? Does anyone else check for consistent and correct reporting?

|  |
| --- |

Description of any facility-level register, log book, or other records collating key obstetric variables at the facility-level. Please include whether this register is paper-based or electronic, when it is updated, and how often it is reported.

|  |
| --- |

Who is responsible for completing the facility-level register?

|  |
| --- |

How often is the facility-level register updated?

|  |
| --- |

How is the information about facility-level key obstetric variables and outcomes currently integrated into audit and feedback?

|  |
| --- |

Who is present during audit and feedback sessions, and who leads the sessions?

|  |
| --- |

Based on the observations and record assessment, what would you consider to be the most appropriate method of implementation of the Robson classification system (manually, using a spreadsheet or automatic calculator, or via electronic records)? Please explain.

|  |
| --- |

Based on the observations and record assessment, what would you consider to be the most appropriate reporting schedule (weekly, bi-weekly, monthly), and why?

|  |
| --- |

Based on the observations and record assessment, who would be the most appropriate champion(s) for implementation?

|  |
| --- |

Any other feedback, observations or reflections

|  |
| --- |
