# Supplementary material for: Feasibility and readiness to implement Robson classification to monitor caesarean sections in public hospitals in Myanmar: Formative research
Source: PLOS Glob Public Health. 2023 Jan 30;3(1):e0001388. doi: 10.1371/journal.pgph.0001388 (PMC10022350; doi:10.1371/journal.pgph.0001388)
Supplement: Attachment — Submitted filename: Responses to reviewers.docx [file pgph.0001388.s004.docx]

**Dear Editors and Reviewers.**

Thank you for the constructive comments, critiques, and suggestions. We have now attempted to address each point and provide a point-by-point response in below. Page and Line numbers are referred to clean manuscript file.

Thank you for your consideration and look forward to hearing from you.

Sincerely,

Dr. Kyaw Lwin Show, on behalf of the authors

**EDITOR**

We invite you to submit a revised version of your manuscript, taking into consideration the comments received from the two reviewers. In particular, to improve overall clarity, please add a table summarizing the barriers and enablers of feasibility and readiness to implement the Robson classification system in public hospitals in Myanmar.

**RESPONSE:**

Thank you. We added a table summarizing the barriers and enablers to implement the Robson classification for better clarity (Table 2).

**EDITOR**

In addition, please ensure that all items in the COREQ checklist are reported in the manuscript.

**RESPONSE:**

Thank you. We ensured that all items in the COREQ checklist are reported in the manuscript and attached COREQ checklist with page numbers as supplementary information.

**EDITOR**

Journal Requirements:

1. Please review your reference list to ensure that it is complete and correct. If you have cited papers that have been retracted, please include the rationale for doing so in the manuscript text, or remove these references and replace them with relevant current references. Any changes to the reference list should be mentioned in the rebuttal letter that accompanies your revised manuscript. If you need to cite a retracted article, indicate the article’s retracted status in the References list and also include a citation and full reference for the retraction notice.

**RESPONSE:**

Thank you. We checked and ensured the reference list is complete and correct.

**EDITOR**

2.  Please insert an Ethics Statement at the beginning of your Methods section, under a subheading 'Ethics Statement'. It must include:

a. (for human participants/donors) - A statement that formal consent was obtained (must state whether verbal/written) OR the reason consent was not obtained (e.g. anonymity). NOTE: If child participants, the statement must declare that formal consent was obtained from the parent/guardian.

**RESPONSE:**

Thank you. We now moved Ethics section to the beginning of Methods section. We also included informed consent obtaining statement as “After informing potential participants about the study, the participants were invited to take part in the study and asked for the written informed consent. All the face-to-face interviews took place in a private room in the study health facilities, such as the break room.” (LINE 102)

**EDITOR**

3.  Please amend your detailed Financial Disclosure statement. This is published with the article. It must therefore be completed in full sentences and contain the exact wording you wish to be published.

a. State the initials, alongside each funding source, of each author to receive each grant.

**RESPONSE:**

Thank you. We added initials of funding receiver alongside with funding source. (LINE 493)

**REVIEWER:**

**Reviewer #1**:

This article addresses an important issue for Myanmar´s maternal care: provider’s perceptions about the feasibility and acceptability to implement a standardized classification system in a context of increasing CS rates to help hospitals monitor those rates. Robson classification system would help them understand which groups contribute most to overall caesarean section rates in order to decide clinical practices for improving clinical management and quality of maternal care.

Minor comments

Plos Global Public health submission guidelines asks to include continuous line numbers in the manuscript file

**RESPONSE:**

We have now added continuous line numbers to facilitate for the review.

**REVIEWER:**

Methods section

Strategy of Analysis: The use of triangulation to strengthen the analysis was not discussed. You mentioned that “We triangulated our qualitative

findings with the assessment by observing the medical records in the facility” (different strategy to study the same problem: different techniques to get the same data)

Did different subjects were involved to answer the same question, different researchers for the same analysis?

**RESPONSE:**

We triangulated and confirmed the findings from qualitative interview with assessment of medical records by direct observation. For example, the respondents said during the interview that variables required for Robson were presented in the patient’s medical records. We confirmed by observing of random patient’s medical records using semi-structured data collection form. We have added the data analysis as follows: “We triangulated the qualitative research findings with medical records in each health facility in order to improve validity of the research findings. We did this by comparing what participants reported as available data in the medical records to what was typically recorded in the observed medical records”. (LINE 174)

**REVIEWER:**

Even though you stated that the analysis was reported according to the Consolidated criteria for reporting qualitative research (COREQ) there are many ítems from this checklist that are not reported in the manuscript. My suggestions is to go through the checklist and the manuscript and include the missing information. For instance: Researcher reflexivity was not discussed, I think that publishing some reflexive content would allow readers to sense how the researcher shaped the entire project, and in particular, the interpretation of findings (Discussion of relationship between researcher and participants during fieldwork; Demonstration of researcher’s influence on stages of research process; Evidence of self-awareness/insight; Documentation of effects of the research on researcher; evidence of how problems/complications met were dealt with)

**RESPONSE:**

Thank you. We ensured that all items in the COREQ checklist are reported in the manuscript and attached COREQ checklist with page numbers as supplementary information.

**REVIEWER:**

Results section

In this verbatim: “Robson classification only might not be useful for us. We do not want that kind of data. We had the audits for reducing caesarean rate, complication sepsis, near misses (abortion), PPH (post-partum hemorrhage) may result after PE (pre-eclampsia). That kind of information is more valuable to us.” (A medical doctor in a tertiary hospital)

I think there is a mistake in “…near misses (abortion)…” Were you referring to NM due to abortion?

**RESPONSE:**

Thank you. We now revised as ‘abortion-related near misses’. (LINE 255)

**REVIEWER:**

I feel that pieces of information are repeated under the way the analysis was organized, and that also it does not follow a connecting thread. The manuscript would benefit from having a table organizing and conceptualizing the findings that might hinder or enable feasibility, acceptability and readiness to implement the Robson classification system in public hospitals across Myanmar.

**RESPONSE:**

Thank you. We added a table summarizing the barriers and enablers to implement the Robson classification for better clarity (Table 2). We have also reviewed for clarity and made revisions throughout.

**REVIEWER:**

**Reviewer #2**:

Dear editor,

thanks for the opportunity of reviewing this article. It seems to me a very good report regarding Robson's Classification, from a qualitative perspective. I think it is the first report using this approach on Robson's Classification. I have some few questions regarding the manuscript, and I believe the manuscript should be accepted for publication.

1) In Methods, authors defined that they would interview professionals from ten different hospitals, 5 tertiary and 5 primary sites of care. However, in results, 76% of participants were from tertiary level referral hospitals. I understand that the sample was not supposed to be representative, according to the study design, however I think authors should justify why they included more professionals from tertiary services.

**RESPONSE:**

Thank you. In Myanmar, there are many Obstetrics and Gynecology specialist doctors in the tertiary and teaching hospitals while only 1-2 Obstetrics and Gynecology specialist doctors in district level hospitals and no specialist doctors at the township level. The structure of the human resources for health explains why many of the respondents were from the tertiary level referral hospitals. In this study, we covered almost all potential administrators and providers from the district/township level hospital. We stated in the methods section as “Obstetrics and Gynecology specialist doctors are available from the district level hospitals and higher” (LINE 120). Furthermore, we considered that tertiary and teaching hospital as entry point to implement Robson classification system in future. We discussed this as “we believe that these hospitals are the entry point for implementing and establishing the use of the Robson classification system because of its resources compared to other hospitals in Myanmar.” (LINE 465)

**REVIEWER:**

2) In page 13, I guess the adequate term is "electronically", instead of "electrically"

**RESPONSE:**

Thank you for pointing this out, we have revised. (LINE 216)

**REVIEWER:**

3) Page 17: is "3 monthly" correct or is it "3 months"?

**RESPONSE:**

Thank you. We have revised to ‘every 3 months’. (LINE 328)
